# Tumor-Primed NK Cells: Waiting for the Green Light

**DOI:** 10.3389/fimmu.2013.00408

**Published:** 2013-11-25

**Authors:** May Sabry, Mark W. Lowdell

**Affiliations:** ^1^Department of Haematology, Royal Free Campus, University College London Medical School, London, UK

**Keywords:** natural killer cells, NK cell dysfunction, cancer immunotherapy, CTV-1, LFA-1, NKG2D

## Abstract

The functional impairment of natural killer (NK) cells has been frequently reported in cancer studies. As one of the central components of host anti-tumor immunity, NK cells exert cellular cytotoxicity against tumor cells, and secrete a cytokine milieu to inhibit tumor progression and enable the recruitment of other immune cells to the tumor site. The unlocking of the full functional potential of NK cells requires successful progression through discrete activation stages that are tightly regulated by a complex array of signaling molecules. Target cell susceptibility to NK cell-mediated killing is dependent on the intensity and specific combination of ligand expression for NK cell receptors. Tumor cells utilize numerous strategies for evading NK cells, including the downregulation of important NK cell-activating ligands. Here, we review key studies on NK cell activation requirements, and argue, based on our findings from NK cell-tumor interactions, that the altered characteristics of tumor-associated NK cells are indicative of unmet signaling requirements for full NK cell activation, rather than NK cell dysfunction in cancer.

## Introduction

Natural killer (NK) cells were first identified in 1975, based on their ability to spontaneously lyse tumor cells in the absence of T and B lymphocytes (1–4). After over 30 years, our understanding of NK cell biology and function lends important insights into their critical role in infection, autoimmunity, hematopoietic stem cell (HSC) transplantation, reproduction as well as tumor immunosurveillance. Besides exhibiting cytotoxicity against a variety of “stressed” cells, NK cells also secrete a milieu of cytokines that participate in shaping adaptive immune responses (5). NK cells share many similarities with cytotoxic T cells (CTLs), including a common progenitor cell, a wide array of cell surface receptors and perforin-dependent killing mechanisms. However, NK cells remain distinct by virtue of their capacity to kill target cells without any prior sensitization or MHC restriction (6). This has led to their wide use in adoptive cancer immunotherapy, despite our incomplete understanding of the regulatory mechanisms and activation requirements that are needed for successful therapeutic approaches. In this review article, we summarize NK cell recognition strategies of tumor targets and the signaling requirements for NK cell-mediated lysis. We also discuss properties of tumor-associated NK cells in light of cancer immune evasion and an unmet activation threshold for NK cell lysis.

## NK Cell Recognition of Tumors

Originally viewed as simple effector cells with a “natural” capacity for killing, NK cells were believed to be the ancestral forerunners of the seemingly more sophisticated T lymphocytes and classed within the innate arm of the immune system. Since then, more sophisticated features that are characteristic of adaptive immunity have been shown to occur in NK cells, including priming, education, and memory (7). Prior to the discovery of NK cell receptors, it was unclear how NK cells could distinguish target cells from normal cells for lysis. The “missing-self” hypothesis was proposed based on the observation that NK cells kill targets with reduced or absent self MHC class I molecules, a phenomenon common to virally infected and transformed cells (8, 9). The subsequent characterization of NK cell inhibitory receptors supported this hypothesis by explaining the molecular mechanisms by which NK cells sensed the downregulation of MHC class I expression (10–19). However, when studies began to show that the absence of MHC class I molecules on tumor cells was insufficient to trigger NK cell lysis, it became clear that our understanding of NK cell target recognition was incomplete (20, 21). As a wide array of activating receptors started to unravel, the “dynamic equilibrium” hypothesis was formulated, postulating that the integration of opposing signals from activating and inhibitory receptors determines the functional outcome of NK cell activity (22).

Recent evidence has shown that when the minimal requirements for NK cell cytotoxicity are met, tumor killing can occur irrespective of the presence of inhibitory signals, which suggests that proponents of the missing-self theory might have been overstating their case. NK cells are negatively regulated by killer Ig-like receptors (KIRs), which bind human leukocyte antigen (HLA)-A, -B, and -C, and C-type lectins, which form CD94/NKG2 receptor complexes recognizing HLA-E (23). The role of HLA-mediated inhibition in regulating NK cell activity is evidenced by studies showing that transfection of appropriate HLA-C alleles into NK susceptible target cells, such as K562, can render them resistant to NK-mediated lysis (24, 25). Additionally, NK-resistant tumors such as the B lymphoma cell line RAJI are known to constitutively express type I and II HLA-C alleles. In the clinical setting, transplantation across HLA barriers has been shown to trigger donor-NK-cell alloreactivity if the recipient lacks KIR-ligands that are present in the donor, which is known as “KIR-ligand mismatch” (26, 27). Studies involving acute myeloid leukemia (AML) patients have demonstrated that KIR-ligand incompatibility can improve survival and engraftment, and reduce the incidence of graft-versus-host disease (GVHD) (20). However, the impact of KIR-mismatch in other clinical settings remains very controversial with several studies showing no advantage of KIR-ligand incompatibility for survival or engraftment (28, 29). Pre-incubation of NK cells with an activating cytokine such as interleukin (IL)-2, resulting in the generation of lymphokine-activated killer cells (LAKs), can result in NK cell killing of targets that were previously resistant. Tumor target cells can also be used to activate NK cells in a manner analogous to IL-2, as we have previously demonstrated using the acute lymphoid leukemia cell line CTV-1, which generated NK cells that are able to lyse NK-resistant tumor cell lines, primary leukemias, and solid tumors, in HLA-matched, allogeneic or autologous settings (30). Moreover, transfection of resistant, HLA-expressing RAJI cells with specific ligands for NK cell-activating receptors renders them susceptible to NK cell lysis (31). Similarly, blockade of certain tumor ligands for activating NK cell receptors on non-HLA-expressing, sensitive K562 cells makes them resistant to NK cell-mediated killing. Thus, providing NK cells with the appropriate combination of activating stimuli unleashes full effector function, such that an NK cell can kill tumor targets even in the presence of strong inhibitory signaling. Building on this knowledge, we aim to further define NK cell activation requirements for tumor killing.

## Tumor Activation of NK Cells

Natural killer cells require the co-engagement of multiple activating receptors in order to exhibit natural cytotoxicity against tumor target cells (32). Work by our group further defined this co-stimulation into two discrete stages, priming and triggering (30). The priming signal can be delivered by an activating cytokine in the tumor microenvironment or a target cell expressing the appropriate intensity and combination of ligands for NK cell activating receptors. The second stage, “triggering,” requires the co-engagement of at least one additional NK cell activating receptor, specific to stressed cells, in order to avoid autoreactivity.

Upon encounter with potential target cells, an immunological synapse forms at the point of contact between the NK cell and the target cell, where NK cell receptors can interact with their respective ligands. Given sufficient activation signals, NK cell cytoskeletal rearrangements are initiated, which result in the polarization of NK cell lytic granules toward the immunological synapse, where they eventually fuse and release their cytotoxic contents on to the target cell (33). In contrast to CTLs, NK cells have their cytotoxic granules preformed before target cell recognition, and so their release is initially constrained until sufficient signaling is achieved (33). NK cells have been shown to establish cytoskeletal polarity more slowly compared to CTLs, and to have a unique sensitivity to minor interference with cytoskeletal dynamics (34).

**Figure 1:**
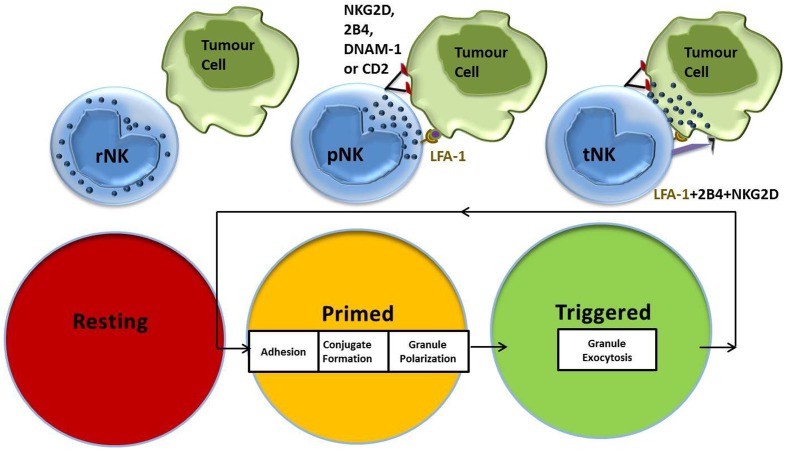
**NK cell activation stages and signaling requirements for natural cytotoxicity**. Resting NK cells require an initial priming signal delivered by an activating cytokine or a target cell expressing the ligands necessary to induce adhesion, conjugate formation, and granule polarization. Co-stimulation of additional NK cell activating receptors by the triggering ligands results in NK cell-mediated cytotoxicity against target cells.

Work by Bryceson et al. showed that the co-engagement of lymphocyte function-associated antigen (LFA)-1 with any of the activating receptors, NK group 2 membrane D (NKG2D), DNAX accessory molecule (DNAM)-1, 2B4 or CD2, is sufficient to establish adhesion, conjugate formation, and granule polarization in NK cells (35, 36). We had previously identified CD2-CD15 interactions as part of the NK priming signal delivered by the leukemic target cell CTV-1, which primes NK cells to kill tumor targets that were previously resistant (31). CTV-1 also expresses ICAM-1, the natural ligand for LFA-1, which has been shown to deliver signals that are crucial for successful NK-target cell conjugate formation (37). This stepwise progression in effector function with specific signaling requirements provides a mechanistic explanation of how the spontaneous capacity of NK cells for killing is regulated (Figure 1). After NK cell-mediated killing of a tumor target cell is achieved, an NK cell is able to restart the activation cycle with the next target cell encounter. IL-2-activated NK cells have the capacity to serially hit up to four target cells (38).

## NK Cell Dysfunction in Cancer: Not a Dysfunction

It is widely thought that cancer patients have immune dysfunctions that are secondary to the presence and progression of their disease (39). Commonly reported NK cell functional impairments in cancer include decreased cytotoxic activity (40–53), downregulation of activating receptor expression (21, 44, 54–56) and intracellular signaling molecules (57–61), defective proliferation (62–64), poor infiltration, decreased cell counts, and defective cytokine production (51, 53, 65). It is important to note that studies since the late 1980s have demonstrated the reversal of these tumor-associated NK cell traits after a few days of *ex vivo* culturing alone or with IL-2, suggesting the absence of any inherent NK cell defect *per se* (66). Instead, we propose that these observations are in fact indicative of a tumor-specific NK cell response, bearing in mind that the tumor itself has undergone selective pressure to grow in an immunocompetent setting.

The weakened capacity of NK cells to kill tumor targets has previously been shown to be “corrected” with the addition of activating stimuli, blockade of inhibitory factors, or when tested against an allogeneic tumor (62, 63). The observation that NK cell-mediated killing of tumor target cells occurs without having undergone any restorative measures is in itself evidence against NK cell functional impairment or incapacity. Loss of CD3-ζ expression is the most frequently cited example of a defective NK cell phenotype and since some of the most important NK cell activating receptors involved in tumor killing are associated with CD3-ζ, including CD16 (67) and several NCRs (61, 68, 69), a generalized loss of function is expected. However, tumor-primed NK cells, which have been shown to have enhanced effector functions, also exhibit marked downregulation of numerous activating receptors (31). More importantly, several studies have reported better killing of tumor targets by NK cell subsets with downregulated receptors such as CD16 or NKp46 compared with their counterparts with normal expression (62, 70). This argues that ligand-induced downregulation of NK cell activating receptors is part of the NK cell response, as has been previously reported (71–74).

Recent studies have highlighted hierarchies in the strength of the activating stimuli required for specific NK cell responses (35, 36, 75). Inside-out signals for LFA-1-dependent adhesion and release of chemokines such as macrophage inflammatory protein (MIP)-1β, exhibit a low threshold for activation, which can be met through the engagement of a single NK cell activating receptor. Degranulation and the release of other cytokines such as tumor necrosis factor (TNF)-α require stronger activating stimuli. Interferon (IFN)-γ displays the most stringent requirements for induction and the highest activation threshold for NK cell receptor cooperation (76). Thus, defective cytokine production by tumor-associated NK cells, which is often reported as a decrease in INF- γ release, can be explained by the absence of sufficient activating signals necessary for its secretion.

Similar to NK cells, tumor-associated T lymphocytes can recognize and eliminate autologous tumors after *ex vivo* culture with IL-2 (60, 77, 78), or anti-CD28 and anti-CD3 mAbs (79), despite their inability to kill those targets *in situ*. Chronic stimulation of T cells, in the absence of a second activation signal has also been shown to decrease T cell receptor expression, proliferative capacity, and responsiveness (80). It can be easily envisaged that chronic stimulation of NK cells such as in an inflammatory/autoimmune disease setting, results in a similar reduction in proliferation and response. Collectively, the observations discussed above argue against an inherent NK cell defect in cancer patients and suggest the absence of sufficient activating signals for full NK cell effector function in the tumor microenvironment. A tumor-primed NK cell, waiting for the second signal to trigger killing, is likely to have downregulated receptors involved in the priming stage, but is still functional and ready for killing upon receipt of secondary stimulation. Interestingly, pre-treatment of tumor cells with histone deacetylase inhibitors, depsipeptide or bortezomib, renders them susceptible to autologous NK cell killing, which suggests that resistance of the tumor target to NK cell-mediated cytotoxicity is determined by tumor-specific gene expression (81).

## Tumor Evasion of NK Cells

The theory of cancer immunosurveillance, as proposed by Burnet and Thomas in 1957 (82), dictates that immune cells continuously monitor the body such that any threat to the immune system is detected and eliminated. Although abandoned shortly after for lack of sufficient experimental evidence (83–86), the subsequent discovery of NK cells led to considerable enthusiasm over the possibility that they function as one of the main effector cells of immunosurveillance (87). Recent studies clearly show the existence of cancer immunosurveillance and support the concept that NK cells play a critical role in tumor control and eradication (88). Evidence for cancer immunosurveillance by NK cells in humans include an 11-year follow-up study of 3500 normal, healthy individuals showing that low NK cell cytotoxicity correlates with an increased risk for cancer (89). The addition of immune evasion as an emerging “hallmark” of cancer, highlights the revival of support for the immunosurveillance theory (90). It is now believed that tumors acquire a set of biological capabilities during their development that allow them to overcome barriers, one of which is likely to be NK cell-mediated anti-tumor immunity. These capabilities are acquired with the help of recruited inflammatory cells and soluble factors in the tumor microenvironment, which play an active role in the process of tumorigenesis.

Early on in the study of NK cell interactions with tumors, Kiessling et al. (39) proposed that cancer evasion of NK cells involves two stages: the early stages of tumor formation and growth are associated with antigen-specific tolerance, whereas the later stages elicit a more generalized state of immunodeficiency. The concept of cancer immunoediting, as introduced by Dunn et al. argues that the immune system plays a role during tumor formation by selecting less immunogenic variants for survival in an immunologically intact environment. Tumors are thus “imprinted” by the immunologic environment in which they form, and only those that have acquired capabilities to evade or suppress immune attack remain. O’Sullivan et al. recently demonstrated that cancer immunoediting by the innate immune system requires NK cell derived IFN-γ, which activates M1 macrophages to function as innate editors (91). Based on evidence for a two-stage hypothesis for NK cell-mediated killing of tumors, we propose that tumors evade NK cell attack directly by lacking either the priming or triggering ligands such that the activation threshold for NK cell granule exocytosis is not met. Once successful evasion of NK cell attack is achieved, the tumor begins to create the microenvironment necessary for its continued growth.

**Figure 2:**
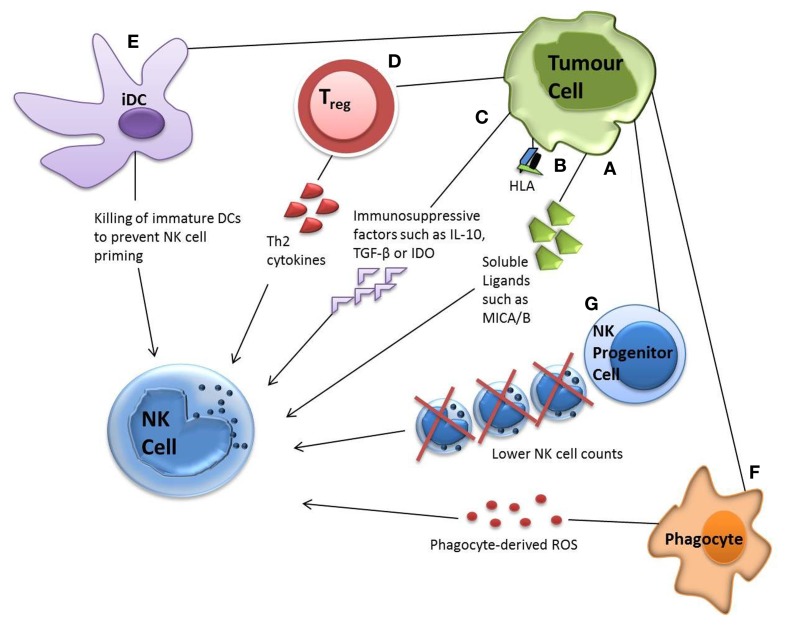
**Tumor evasion strategies**. Tumor cells can evade NK cell attack via direct or indirect mechanisms. Direct mechanisms include **(A)** shedding soluble ligands for NK cell activating receptors **(B)** upregulation of HLA molecules and **(C)** release of inhibitory cytokines. Indirect mechanisms include **(D)** activation of inhibitory regulatory T cells **(E)** dendritic cell killing and **(F)** phagocyte-derived inhibitory cytokines. Tumor cells have also been shown to decrease the number of NK progenitor cells **(G)**, hence lowering NK cell counts.

Direct evasion of NK cells by tumor targets can be accompanied by various other escape mechanisms. For example, tumors have been shown to minimally express or shed ligands for important NK cell receptors, such as NKG2D ligands UL16-binding protein 2, major histocompatibility complex (MHC) class I chain-related molecules A and B molecules (MICA/MICB). They have also been reported to upregulate MHC class I, soluble MIC and FasL expression in order to increase inhibitory signaling (21, 92–94). The release of immunosuppressive factors such as IL-10, TGF-β, and indoleamine 2,3-dioxygense (IDO) by tumor targets has also been reported, which can suppress the adaptive anti-tumor immune response or skew the immune response toward a Th2 response with significantly less anti-tumor capacity (95–99) (Figure 2).

Indirect mechanisms for NK cell evasion by tumors can involve numerous cell types from the immune system. Recruitment of inflammatory cells that are actively immunosuppressive has been demonstrated, including regulatory T cells (Tregs), myeloid-derived suppressor cells (MDSCs), and phagocytes secreting reactive oxygen species (ROS) (100). Some tumors alter their expressions of IL-6, IL-10, vascular epithelial growth factor or GM-CSF, impairing dendritic cell function and maturation, hence NK cell priming. Tumor growth has also been shown to decrease NK cell count by reducing the numbers of its lymphoid progenitor (101) (Figure 2).

## Conclusion – Moving Forward

Studies summarized here argue against inherent NK cell defects in cancer, based on their retained capacity for effector functions as well their differential activation profiles in response to varying stimuli. NK cell functional responses can be anti-tumoral, anti-viral, or immunomodulatory, depending on the type of threat faced by the immune system and the activating NK cell signals received. Studies over the past decade have shown significant differences in specific NK cell responses according to the type of stimulus, be it an infected cell, a transformed cell or an exogenous cytokine. This should be taken into consideration in the application of strategies involving *ex vivo* culture of NK cells to enhance NK cell functional properties. In the case of cancer immunotherapy, studying tumor-specific responses of NK cells should be the focal point for better specificity and efficacy of treatments. Further defining NK cell activation stages as coupled by their requirements for receptor cooperation is critical, since it is clear that the entire answer does not lie in KIR-mismatch and the overcoming of inhibitory signaling. A clear understanding of NK cell activation requirements at the bench may lead to novel therapeutic strategies for the treatment of cancer.

## Conflict of Interest Statement

Mark W. Lowdell is a consultant to Coronado Biosciences, which has licensed the patent to clinical commercialization of tumor-primed NK cells. The other co-author declares that the research was conducted in the absence of any commercial or financial relationships that could be construed as a potential conflict of interest.
